# Sarcoidosis: Various Presentations, Coexisting Diseases and Malignancies

**DOI:** 10.7759/cureus.16967

**Published:** 2021-08-07

**Authors:** Mrunanjali Gaddam, Ugochi Ojinnaka, Zubayer Ahmed, Amudhan Kannan, Huma Quadir, Knkush Hakobyan, Jihan A Mostafa

**Affiliations:** 1 Internal Medicine, California Institute of Behavioral Neurosciences & Psychology, Fairfield, USA; 2 Internal Medicine, Andhra Medical College, Visakhapatnam, IND; 3 Family Medicine, California Institute of Behavioral Neurosciences & Psychology, Fairfield, USA; 4 Medicine, Jawaharlal Institute of Postgraduate Medical Education and Research, Puducherry, IND; 5 General Surgery Research, California Institute of Behavioral Neurosciences & Psychology, Fairfield, USA; 6 Internal Medicine/Family Medicine, California Institute of Behavioral Neurosciences & Psychology, Fairfield, USA; 7 Neurology, California Institute of Behavioral Neurosciences & Psychology, Fairfield, USA; 8 Diagnostic Radiology, California Institute of Behavioral Neurosciences & Psychology, Fairfield, USA; 9 Adjunct Faculty, California Institute of Behavioral Neurosciences & Psychology, Fairfield, USA

**Keywords:** sarcoidosis, pathology, association, thyroid, other diseases, malignancy, complex presentation

## Abstract

Sarcoidosis is a rare, chronic inflammatory disease with a characteristic non-caseating granuloma formation. It affects women more than men. The lung is the most commonly affected organ, however, extrapulmonary involvement is also seen. Sarcoidosis can affect any organ or tissue and can also involve multiple organs simultaneously. As a disease, it shares clinical symptoms with a variety of autoimmune, non-autoimmune disorders and malignancies. Not only it mimics clinically, but it also coexists with these diseases, posing a significant diagnostic challenge. During this literature review, we obtained data from the previously published PubMed articles within the last five years and reviewed the possible etiological association and clinical coexistence between sarcoidosis and other diseases/malignancies. We aimed to determine the common clinical manifestations, various complex presentations of sarcoidosis and pathophysiological considerations for the association, and to emphasize the link with other diseases, particularly thyroid disorders/malignancies. Physicians should be aware of these associated diseases and should always make a clinical suspicion when confronting a sarcoidosis patient. Thus, a comprehensive diagnostic evaluation for these associated conditions ought to be done in sarcoidosis patients to avoid any delay in the curative treatment for these coexisting diseases and to prevent substandard outcomes.

## Introduction and background

Sarcoidosis is a chronic multisystem granulomatous inflammatory disease of unknown etiology. The prevalence of sarcoidosis ranges from 2.2 to 160 cases per 100,000 people [1]. European countries have a high prevalence rate, mainly Scandinavia with the world’s highest annual incidence rate (64 cases per 100, 000 people) [2,3], Asian people such as the Chinese or Japanese, are less prone to this disease [4]. It most commonly affects young adults aged 25-45 years with female predominance [2]. In more than 90% of the patient's lungs are affected, and 30%-50% have extrapulmonary manifestations [5,6]. Although the exact mechanism of sarcoidosis is unknown, recent studies have reported that in a genetically predisposed individual T-cells mediates an immune response to self or foreign antigens and lead to granuloma formation.

Most common clinical manifestations of sarcoidosis are non-specific generalized symptoms like fever, fatigue, myalgias, arthritis which is also seen in various autoimmune disease that need to be differentiated from sarcoidosis. It may manifest acutely as Lofgren syndrome (a triad of erythema nodosum, bilateral hilar lymphadenopathy, and arthritis). Pulmonary sarcoidosis usually presents nonspecifically as dry cough, dyspnea and it progresses over stages.

· Stage 0: no pulmonary infiltrates or lymphadenopathy

· Stage 1: hilar and mediastinal lymphadenopathy alone

· Stage 2: lymphadenopathy and pulmonary infiltrates

· Stage 3: pulmonary infiltrates alone

· Stage 4: pulmonary fibrosis

Extrapulmonary locations are skin, lymph nodes, eye, liver, spleen, heart, nervous system, kidney, parotid gland, nose, larynx, bones, skeletal muscles, genitourinary tract, gastrointestinal tract [6]. Although rare, endocrine gland (e.g., thyroid, pituitary, adrenal) involvement causing functioning abnormalities are also reported causing a great clinical significance [7].

Owing to its multiple organ involvement, its various presentation which could mimic other organ diseases and also its coexistence with several endocrine, non-endocrine autoimmune conditions and malignancies, the diagnosis could be challenging. Sarcoidosis of the thyroid gland was first reported in 1938 and the autopsy showing granuloma confirmed it in a 51-year-old male patient [8]. Fallahi et al. observed that sarcoidosis is one of the 10 most common autoimmune diseases observed in patients with autoimmune thyroiditis [9]. From the study in Asia, Wu et al. reported that sarcoidosis patients have autoimmune thyroid disease, Sjogren’s syndrome and ankylosing spondylitis more compared to healthy people [10]. Sarcoidosis can present coexisting with malignancy. Spiekermann et al. reported 59 cases of sarcoidosis with coexisting cancers with breast cancer as most common followed by thyroid cancers [11]. In this literature review, we aimed to describe the immune pathophysiology of sarcoidosis, sarcoidosis involving various organ systems and its presentations, and to study the possible mechanism and associations with other coexisting diseases, malignancies, and the influence of these co-morbidities. 

## Review

Pathogenesis of sarcoidosis and pathophysiological considerations for multisystemic nature and coexistence with other conditions (autoimmunity and immunological association with other diseases)

Pathogenesis of Sarcoidosis

**Figure 1:**
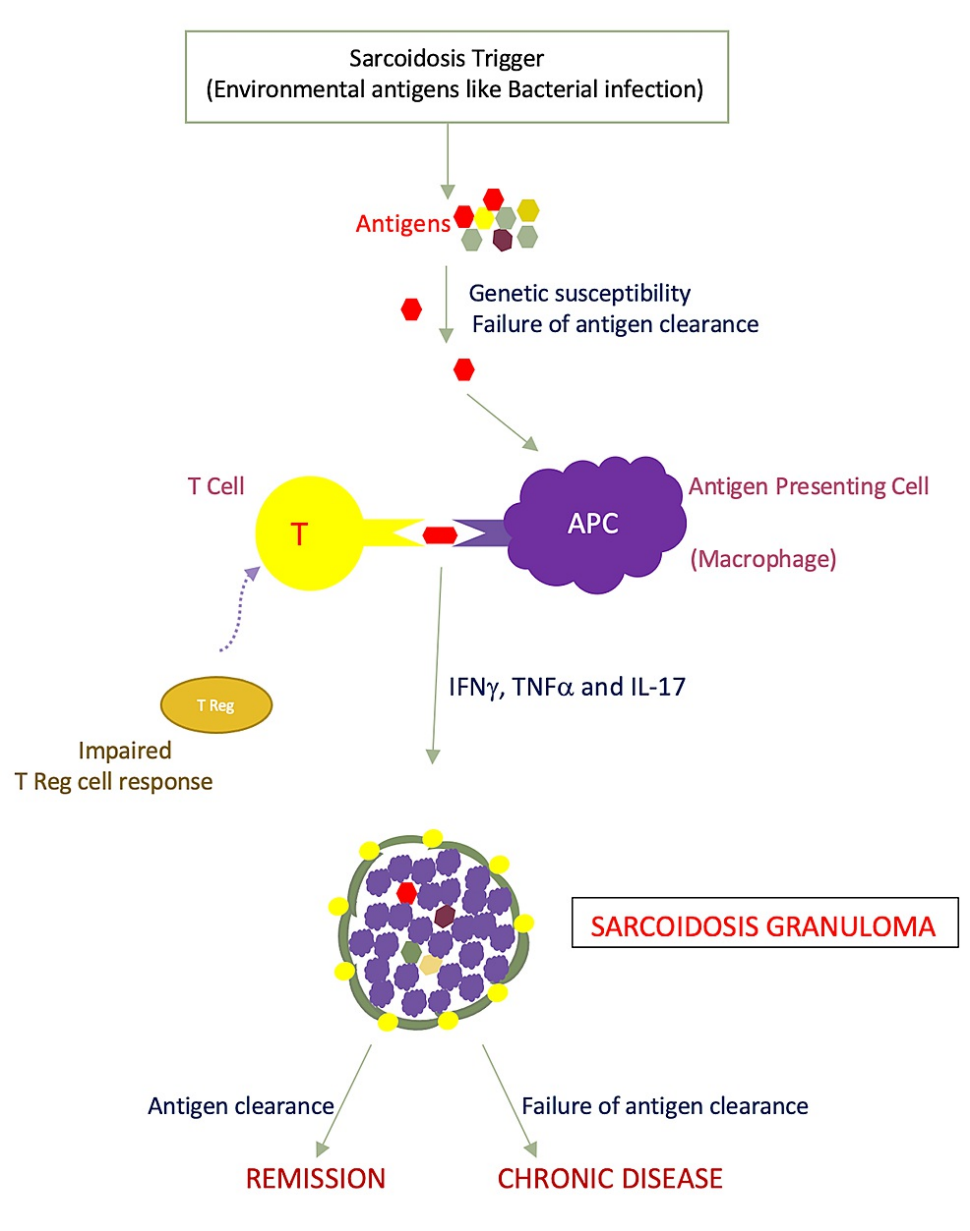
Pathogenesis of Sarcoidosis Granuloma T Reg cells - Regulatory T-cells, IFNγ - Interferon gamma, TNFα - Tumor necrosis factor alpha, IL-17 - Interleukin-17

Even though science is growing, the exact cause of sarcoidosis is still unclear. Environmental antigen, bacterial or other is assumed to be the potential triggers. In a genetically susceptible individual, the causing antigen is not successfully eliminated leading to a granulomatous inflammation [12]. An airborne antigen can be the cause for the lung being the most affected organ than other organs [12]. Interaction between T lymphocytes and macrophages can lead to granuloma formation [12]. Primarily, pulmonary sarcoidosis is considered a T-cell mediated disease. Loss of tolerance to self-antigen on T-cell has been reported even though clear evidence of autoimmunity is lacking. In sarcoidosis patients, human leukocyte antigen DR type (HLA-DR)-bound peptides of vimentin were obtained in the bronchoalveolar lavage (BAL) [13]. In a recent study, Grunewald et al. suggested T-cell mediated autoimmunity by reporting accumulation of presumably vimentin specific CD4+ T-cells in the lungs of HLA-DRB1*03 positive sarcoidosis patients [14]. Vihlborg et al. in a prospective cohort study conducted in Sweden reported that increased incidence rate of sarcoidosis (3.94) and rheumatoid arthritis (2.94) in male workers who were exposed to silica dust, suggesting a probable immunopathogenesis either to antigen exposure or immunomodulation in a susceptible patient population [15]. T-cells that mediate the production of cytokines, chemokines, and various cellular immunological and inflammatory responses are seen in large numbers in the granulomatous process of the disease [16]. In conclusion, failure to eliminate the foreign antigen or loss of tolerance to self-antigens in a genetically susceptible individual or in a certain patient population can lead to sarcoidosis development (Figure 1).

Pathogenesis of Sarcoidosis as a Multisystem Disorder

Studies reported the importance of T helper 17 (Th17) cells in sarcoidosis pathogenesis [16]. The interleukin (IL)-17+/CD4+ lymphocytes can induce an exacerbated inflammatory response in the body. The multisystemic nature of the disease is supported by the evidence of an increased number of Th17 cells in the lung and the peripheral blood of active sarcoidosis patients. Even the sarcoid alveolar macrophages produce increased amounts of IL-17.

Pathology of Sarcoidosis Association With Other Disorders/Malignancy

Sarcoidosis doesn’t meet the criteria for autoimmune diseases; however, the co-relation between sarcoidosis and autoimmune diseases is reported frequently [17]. An increase in humoral response and hyperglobulinemia is seen frequently in sarcoidosis, resulting in increased autoimmune co-morbidities [10,17,18]. Several endocrine and non-endocrine autoimmune diseases may coexist with sarcoidosis [19].

The underlying mechanism between the coexisting of sarcoidosis and malignancy is still unclear [20]. Longstanding inflammation and immune dysregulation may be risk factors for developing malignancy [21]. Release of some inflammatory cytokines, activation of Th1/Th17 cells, decreased regulatory T-cells and impaired CD4 T-cells, may promote the proliferation of cells and blood vessels, tissue remodeling, and stromal growth leading to a tumor [22].

Clinical features of sarcoidosis

Lofgren’s syndrome, a triad of bilateral hilar lymphadenopathy, arthritis, and erythema nodosum is an acute presentation of sarcoidosis. Constitutional symptoms like fever, malaise, fatigue, myalgias, weight loss are seen frequently [6]. Lung involvement is frequent and presents as dry cough, dyspnea, and chest pain. Various extrapulmonary manifestations are seen. Skin manifestations such as erythema nodosum, papular or plaque-like or nodular lesions, lupus pernio are seen. Ocular manifestations like keratoconjunctivitis sicca, uveitis are seen. Cardiac manifestations like pericarditis, myocarditis, conduction blocks are seen. Vasantrao et al. reported a case of pericardial sarcoidosis presenting as pyrexia of unknown origin [23]. Sometimes exocrine gland or lymph node enlargement may be the initial sign [24]. Less frequently affects liver, spleen, heart, brain. Sarcoidosis may also present with multiple endocrine manifestations like hypopituitarism or thyroid involvement [25]. Central diabetes insipidus is often the presenting symptom in patients with hypothalamus-pituitary axis involvement [26]. Laboratory findings like hypercalcemia and elevated angiotensin-converting enzyme (ACE) levels can also be seen as the only presentation. Raised ACE levels have less sensitivity for diagnosis; however, in patients who have high ACE levels at the initial presentation, it is useful for following disease course [27]. Biopsy of the most accessible organ and histopathological examination of non-caseating granulomas confirms the diagnosis [12]. Most frequently involved organ systems such as lungs, skin, joints, eyes, parotid glands, kidneys with symptoms like kerato-conjunctivitis sicca, weight loss, lymphadenopathy, fever, cutaneous and pulmonary complaints overlap with various autoimmune disease symptoms [28]. Therefore, a systematic approach is required for the diagnosis of sarcoidosis as diagnosing by history and physical examination alone is difficult as it mimics various other diseases, malignancies and histopathological study should be done for the presence of sarcoid granulomas to confirms the diagnosis.

Diseases that mimic or coexist with sarcoidosis

Various case-control studies, cohort studies, case reports, and case series reported sarcoidosis with another coexisting autoimmune disease or as mimicking malignancy. Nevertheless, these reports are useful for physicians, to be aware that various systemic diseases may coexist with sarcoidosis [28]. A Taiwan case-control study conducted by Wu et al. comparing 1237 sarcoidosis patients with 4948 age and sex-matched healthy controls found that autoimmune co-morbidities are more in sarcoidosis patients (17.6%) than in controls (9.4%, P < 0.05). Autoimmune thyroid disease, Sjogren’s syndrome, and ankylosing spondylitis are the most frequently demonstrated autoimmune diseases in sarcoidosis patients and are mostly seen after the diagnosis of sarcoidosis. The sex-stratified analyses demonstrated a remarkable association of ankylosing spondylitis and sarcoidosis in both sexes, however, Sjogren’s syndrome is seen more in females and autoimmune thyroid disease is seen more in male patients respectively [10]. Sarcoidosis occurring concurrently with diseases like systemic lupus erythematosus and amyloidosis is also reported in the literature [29]. Some of the articles that we used for this review showing the association between sarcoidosis and other diseases are summarized in Table 1.

**Table 1:** Few diseases (autoimmune or non-autoimmune) associated with sarcoidosis are reported in the literature and summarized in this review

Author(s)	Ethnicity	Type of study	Reported associated diseases
Rezgui et al. (2016) [29]	-	Case Report	Systemic lupus erythematosus, Amyloidosis
Alsahwi et al. (2016) [25]	African American	Case Report	Non-parathyroid hormone-mediated hypercalcemia, sarcoidosis of thyroid gland and hypopituitarism
Wu et al. (2017) [10]	Taiwan	Case-Control Study	Autoimmune thyroid disease, Sjogren’s syndrome, Ankylosing spondylitis, Systemic lupus erythematosus, Myasthenia gravis, Systemics sclerosis, Psoriasis, Rheumatoid arthritis, Dermatomyositis, Vitiligo, Alopecia areata
Dayal et al. (2017) [30]	Mixed ethnic origin	Case Report	Autoimmune thyroiditis, Diabetes mellitus, Pancreatitis
Terwiel et al. (2019) [31]	Multiple Ethnicity	Review Article	Autoimmune thyroid disease, Ankylosing spondylitis, Rheumatoid arthritis, Sjogren’s syndrome, Systemic lupus erythematosus, Psoriasis, Vitiligo, Vitiligo, multiple sclerosis, Crohn's disease, ulcerative colitis, celiac disease, autoimmune hepatitis, primary sclerosing cholangitis

Sarcoidosis and Thyroid

Various thyroid disorders like goiter, subacute thyroiditis, thyroid cancer, and sarcoid infiltration of the thyroid are seen in sarcoidosis patients. Several pieces of literature reported the co-occurrence of sarcoidosis and autoimmune thyroiditis (AT). Out of 348 sarcoidosis patients evaluated by Isern et al., 10 had AT, with nine cases having sarcoidosis precede AT [32]. Papadopoulos et al. reported thyroid autoimmunity in 13 patients among 78 sarcoidosis patients (six with Hashimoto thyroiditis, five with isolated thyroid serology positive, two with Graves’ disease) [19]. In another study, Nakamura et al. reported 17 cases of thyroid autoimmunity in a series of 62 pulmonary sarcoidosis patients [33]. Yamamoto et al. reported a case of cutaneous sarcoid in a Hashimoto thyroiditis patient [34]. Semiz et al. reported a case of sarcoidosis and Hashimoto thyroiditis coexistence in an 80-year-old female who presented with Lofgren’s syndrome triad of symptoms. On laboratory evaluation, ACE levels are raised, thyroid function tests showed low free T3 and free T4, high thyroid-stimulating hormone (TSH), raised anti-thyroperoxidase (anti-TPO), and anti-thyroglobulin (anti-TG) antibodies. Computed tomography (CT) showed multiple mediastinal and hilar lymphadenopathy. Thyroid ultrasound (USG) showed diffuse thyroiditis. A diagnosis of sarcoidosis (Lofgren’s syndrome) and Hashimoto thyroiditis is made and treated with levothyroxine and prednisolone [35]. In another case report, Makino et al. reported that the exacerbation of sarcoidosis can induce an immunological storm leading to thyroid autoantibodies production causing Graves’ disease [36]. The prevalence of Hashimoto’s thyroiditis is high (3-11%) compared to other thyroid diseases in patients with sarcoidosis [33]. Even, Muzaffar et al. reported that the prevalence of these antibodies and Hashimoto’s thyroiditis is high in sarcoidosis patients when compared to sex and age-matched control group [37]. In an observational study of thyroid samples, Guerlain et al. reported a case of sarcoidosis presenting as a thyroid calcification [38]. Studies showing the thyroid and sarcoidosis association as summarized in Table 2. In conclusion, clinicians should be aware of the frequent association between sarcoidosis and thyroid autoimmunity and should evaluate thoroughly all sarcoidosis patients for thyroid disease especially thyroid antibodies for autoimmune thyroiditis or any other thyroid conditions.

**Table 2:** Few pieces of literature reporting sarcoidosis and thyroid autoimmunity association

Author(s)	Type of study	Number of patients with sarcoidosis	No of sarcoidosis patients reported thyroid autoimmunity
Isern et al. [32]	Case series	348	10
Papadopoulos et al. [19]	Case series	78	13 (six with Hashimoto thyroiditis, five with isolated thyroid serology positive, two with Graves’ disease)
Nakamura et al. [33]	Case series	62	17
Yamamoto et al. [34]	Case report	1 (cutaneous sarcoidosis)	Hashimoto thyroiditis
Semiz et al. [35]	Case report	1 (Lofgren’s syndrome)	Hashimoto thyroiditis
Makino et al. [36]	Case Report	1	Graves’ disease later converted to hypothyroidism

In a national level questionnaire-based study conducted on 3835 self-reported sarcoidosis patients, they compared cases with and without self-reported hypothyroidism among sarcoidosis patients. They educated that hypothyroidism is a prevalent coexisting disease in sarcoidosis patients and sarcoidosis patients with hypothyroidism have more multiple organ presentations (51% compared to 44%, p < 0.001), such as skin, joints, eyes, liver, lacrimal glands showing sarcoid presentations than typical pulmonary sarcoidosis. Among various presentations, cutaneous sarcoidosis presenting as erythema nodosum, lupus pernio, papules, plaques, skin pigmentation, and subcutaneous nodules around scars and tattoos is most common in the hypothyroid group. Among these self-reporting hypothyroid groups, depression, fatigue, and physical impairment are reported significantly compared to the non-hypothyroid sarcoidosis group of patients [39]. In conclusion, every sarcoidosis patient, mainly in patients with multi-organ presentations should be screened for hypothyroidism and it might be a possible treatable contributor to depression, fatigue, and physical impairment in those patient populations.

In another retrospective study conducted on 317 patients in Japan, Kinoshita et al. reported dilated cardiomyopathy and cardiac sarcoidosis as risk factors for the development of amiodarone-induced hyperthyroidism [40]. In brief, sarcoidosis can also act as an independent risk factor for hyperthyroidism development. So, physicians should diagnose and treat it thoroughly to prevent new disease onset especially thyroid disorders.

In a prospective study, conducted in an outpatient clinic for the prevalence of other autoimmune diseases in 3069 autoimmune thyroiditis patients (AT) versus two age and sex control groups (1023 subjects from a general population and 1023 subjects with non-toxic multinodular goiter), Fallahi et al. reported a very high prevalence of other autoimmune diseases in AT patients compared to controls. The most frequently observed combination of diseases was AT along with chronic atrophic gastritis (CAG) and vitiligo, and AT along with CAG and polymyalgia rheumatica. Sarcoidosis is seen in 19 patients of AT while only in two patients in the controls group (P = 0.0133) [9]. In their other literature, they reported the same association of autoimmune diseases even in Graves’ disease patients and sarcoidosis is reported in 17 Graves’ disease patients when compared to one in the control group [41]. In conclusion, physicians should be cautious about these associated diseases including sarcoidosis and evaluate if AT or Graves’ disease is not getting controlled, or any non-specific symptoms arise newly to avoid any hindrance in the diagnosis.

Not only just the thyroid, diverse autoimmune and non-autoimmune endocrine disorders can be seen concurrently with sarcoidosis, although very few cases were reported making a diagnostic challenge to the physicians. Dayal et al. reported a case of a 9-year-old girl with probable sarcoidosis with autoimmune thyroiditis, diabetes mellitus, and hypercalcemic pancreatitis [30]. Alsahwi et al. reported a case of sarcoidosis with multiple endocrine manifestations occurring simultaneously in a 36-year-old African American woman that include non-parathyroid hormone-mediated hypercalcemia, sarcoidosis of thyroid gland and hypopituitarism (secondary amenorrhea, secondary adrenal insufficiency, growth hormone deficiency) [25]. Therefore, clinicians should be very cautious in patients with multi-organ disease and should keep sarcoidosis in mind and evaluate for it as sarcoidosis can involve any organ and can mimic various organ diseases causing a diagnostic challenge.

Some variable presentations of sarcoidosis

Miliary Sarcoidosis

Although very rare, miliary sarcoidosis is reported in the literature [42]. So, in evaluating the miliary pattern, after ruled out infections like tuberculosis (TB), Histoplasmosis, coccidioidomycosis, and hematogenous metastasis (thyroid, renal, breast), clinicians should evaluate for sarcoidosis which will be confirmed by the presence of non-caseating granulomas in a transbronchial lung biopsy.

Neuro Sarcoidosis

Neuro sarcoidosis is seen in 5-26% of sarcoidosis patients [43]. Even though rare, isolated hypothalamic-pituitary axis involvement can also be seen. Hassani et al. reported a case of pituitary sarcoidosis in a 41-year-old woman post thyroidectomy imitating as pituitary adenoma with more complications than systemic sarcoidosis [44]. In conclusion, clinicians should do long-term multidisciplinary management in patients with pituitary sarcoidosis for a better outcome and prevent complications.

Orbital Sarcoidosis 

Even though uveitis is the most common presentation of sarcoidosis we can also see orbital and adnexal (ocular muscles, lacrimal glands, eyelids) involvement with the extraocular muscles involvement most commonly. In a retrospective case study conducted on 59 patients with non-thyroid extraocular muscle enlargement by Savino et al., four were diagnosed with sarcoidosis involving the medial rectus and superior rectus presenting as a mass effect [45]. Two cases, one with bilateral lacrimal gland enlargement and the other with neurosarcoidosis with optic nerve involvement were reported by McNab [46]. Kim et al. presented two other cases with active sarcoid orbitopathy that were initially misdiagnosed as thyroid eye disease [47]. Even it can also present as sarcoid myositis of extraocular muscles with sometimes leading to vision loss [48]. In patients with orbital manifestations alone, the systemic disease can develop in up to 8% of them in the next five years [49]. In conclusion, in patients with unusual orbital manifestations or poor response to treatment, clinicians should keep sarcoidosis in mind as one of the broad differential diagnoses and should evaluate for it with an orbital biopsy for accurate diagnosis, as vision loss can occur in some patients if go untreated.

 *Multi-Organ Sarcoidosis*

Not only single organ involvement but complex presentations involving various organs simultaneously is also seen in sarcoidosis. Wadhwa et al. reported a case of splenic, hepatic, and thyroid sarcoidosis presenting as organomegaly diagnosed by autopsy along with autoimmune thyroiditis [50]. Another case of isolated hepatosplenic sarcoidosis presenting as hypercalcemia without lung involvement in a patient with papillary thyroid cancer (PTC) post thyroidectomy was reported by Haykal et al. [51]. In conclusion, atypical sarcoidosis involving various other organs excluding the lung is very challenging. Laboratory, imaging, and histopathological evaluation should be done for an accurate diagnosis promptly.

Malignancies coexisting with sarcoidosis

The relationship between malignancy and sarcoidosis is not clear yet. In a prospective cohort study, Brincker and Wilbek reported malignancies more in the sarcoidosis patients compared to the general population (OR = 1.42, p <0.02) [52]. A large retrospective cohort study was done on 9015 sarcoidosis patients, reported that these people have an increased risk of malignancies (relative risk = 1.3, p <0001) when compared to age, sex-matched population incidence rates [53]. On contrary, in another cross-registry study of 555 danish sarcoidosis patients, no increase incidence of malignancy is found, although this might slightly be due to lack of power [54]. Although breast, thyroid cancers are the most common [11] sarcoidosis coexisting with various other cancers were reported in the literature [55].

Sarcoidosis With Papillary Thyroid Cancer

Riis et al. reported a case of sarcoidosis with coexisting PTC presenting with clinical symptoms of moderate hypercalcemia [56]. Presentation of sarcoidosis and PTC simultaneously will be a great diagnostic challenge as both conditions involve the lymph nodes of the neck. Poplawska-Kita et al. reported a case of thyroid cancer in a 68-year-old female who died of sudden cardiac arrest. PTC with metastasis to sella turcica and associated sarcoidosis of heart, lung, hilar, and mediastinal lymph nodes is revealed on autopsy [57]. In conclusion, clinicians should be aware of the heterogenicity of PTC and must be skeptical and should be promoted in the diagnosis of metastasis involving various organs by doing a thorough diagnostic workup, and the biopsy of the lymph nodes will confirm the other accompanying diseases.

Sarcoidosis With Other Cancers

Not only PTC, but sarcoidosis can also cause a diagnostic dilemma by resembling in presentation with various other cancers or coexisting with other cancers. Bala et al. reported a case of testicular sarcoidosis mimicking as testicular malignancy in a 49-year-old Indian male who also has coexisting PTC [58]. Although rare, a case of cervical cancer in a 31-year-old female presenting as cervical fungating polyp with thoracic lymphadenopathy and without pelvic and para-aortic lymph node involvement which on further workup confirmed sarcoidosis coexistence [55]. In conclusion, whenever a lymph node pattern that is not in correlation with the cancer is seen, diagnostic uncertainty arises. So, clinicians should do a histopathological confirmation for an accurate and early diagnosis and should keep sarcoidosis in mind.

Co-morbidities on mortality of sarcoidosis

The coexisting diseases have a strong influence on mortality in patients with sarcoidosis. In an observational prospective cohort study conducted by Nowinski et al. on 557 sarcoidosis patients and non-sarcoidosis patients as controls thyroid diseases are seen more frequently in the sarcoidosis group compared to controls. The study reported that the more the number of co-morbidities the less the survival probability [59].

Limitations

We faced few limitations while conducting this traditional review. Many studies used in this review had small sample sizes (case reports). Only articles published in the last five years in PubMed were included. Articles in languages other than English could not be included.

## Conclusions

We conducted this literature review to discover more about the complex nature of sarcoidosis disease. Here we elaborate on the pathogenesis underlying the sarcoidosis, its multisystemic nature and its association with various other disease conditions, its variable presentations and its coexistence with various autoimmune, non-autoimmune disease and malignancies. In this article, we tried to mention all the coexisting diseases and malignancies that we encountered in the last five years of PubMed articles.

This literature review would be beneficial to the clinicians to have a better understanding of the sarcoidosis disease which will help them to diagnose the disease at an early stage, and to do evaluations systematically for other commonly associated diseases and to treat the patients thoroughly on time without any lacking, ultimately preventing the worst scenarios and the complications. As only a few papers that we used in our review article are of great sample size there is a need to study more about sarcoidosis complexity specifically in the larger group for better insight, especially in patients with various other associated diseases.
